# Ethical Leadership as Antecedent of Job Satisfaction, Affective Organizational Commitment and Intention to Stay Among Volunteers of Non-profit Organizations

**DOI:** 10.3389/fpsyg.2018.02069

**Published:** 2018-11-16

**Authors:** Paula Benevene, Laura Dal Corso, Alessandro De Carlo, Alessandra Falco, Francesca Carluccio, Maria Luisa Vecina

**Affiliations:** ^1^Department of Social Sciences, Libera Università Maria SS. Assunta, Rome, Italy; ^2^Dipartimento di Filosofia, Sociologia, Pedagogia e Psicologia Applicata, Università degli Studi di Padova, Padova, Italy; ^3^Departamento de Psicología Social, del Trabajo y Diferencial, Universidad Complutense de Madrid, Madrid, Spain

**Keywords:** non-profit organizations, job satisfaction, affective organizational commitment, volunteers’ turnover, volunteers’ management, non-profit management, volunteering, ethical leadership

## Abstract

The aim of this paper is to investigate among a group of non-profit organizations: (a) the effect of ethical leadership (EL) on volunteers’ satisfaction, affective organizational commitment and intention to stay in the same organization; (b) the role played by job satisfaction as a mediator in the relationship between EL and volunteers’ intentions to stay in the same organization, as well as between EL and affective commitment. An anonymous questionnaire was individually administered to 198 Italian volunteers of different non-profit organizations. The questionnaire contained the Ethical Leadership Scale, the Volunteers Satisfaction Index, the Affective organization Scale, as well as questions regarding the participants’ age, sex, type of work, level of education, length of their volunteer works, intention to volunteer in the following months in the same organization. The construct as well the effects of EL on volunteers is approached in light of the Social Exchange Theory and the Social Learning Theory. Structural equation models were used to test hypothesized relationships. The results confirm the role of mediation of volunteer satisfaction in the relationships between the variables studied. In particular, EL was found to be positively associated both with volunteers’ intention of staying and with their affective commitment. In the first case this relationship is fully explained by the mediation of the volunteers’ satisfaction, while the latter is explained by both direct and indirect factors. To the authors’ knowledge, this the first attempt to understand the role played by EL on volunteers’ behavior and, more in general, in the management of non-profit organizations. Findings are relevant both for practitioners and managers of non-profit organization, since they suggest the relevance of the perception of EL by volunteers, as well as for scholars, since they further deepen the knowledge on EL and its effects on the followers. Limits of the study: the questionnaire was administered only among a group of non-statistical sample of volunteers. Furthermore, the study reached only volunteers from Italian non-profit organization.

## Introduction

Scandals involving Endrom and WorldCom in the United States, the Banca Popolare di Lodi in Italy, or even Volkswagen in Germany, have brought ethical questions about these organizations’ corporate management under the attention of the media, the broader public, and even the academia (Carson, 2003). As a consequence, over the past two decades a number of studies have been developed on the ethical management of for-profit organization (see e.g., Coffee, 2005; Agrawal and Cooper, 2017; Zyglidopoulos et al., 2017). These studies have highlighted the need for a strong commitment on behalf of senior managers in order to leverage the ethical behavior of their organizations. In fact, senior managers play a pivotal role not only in taking ethical choices but also in modeling and aligning the behaviors of the middle management and the employees in this direction (Treviño et al., 2000; Lutz Allen et al., 2013). Moreover, the effects of ethical leadership (EL) in organizations is not only limited to the need to prevent damages to a firm’s public image. In fact, EL has proven to play a significant role in the generation of positive attitudes and behaviors among the members of an organization, which in turn are important antecedents of high levels of individual and organizational performance (Rowold and Rohmann, 2009; Lam et al., 2016; Ren and Chadee, 2017). This explains the growing interest in the role played by EL in organizations.

As a matter of fact, the relevance of EL in the management of organizations is not limited to for-profit entities. Ethical management has emerged as a crucial factor also for attracting human and financial resources among non-profit organizations (NPOs), especially after the scandals that in recent years have affected the non-profit world as well (Grunewald, 2008).

However, to the authors’ knowledge, the role played by EL among NPOs has not yet been explored to date. This is an important gap to address, especially in light of EL’s positive organizational outcomes, such as the attractiveness of the organization on possible future employees (Strobel et al., 2015), which are critical for NPOs, as well as EL’s negative correlation with employees’ intention to quit the organization. The latter relationship – both direct and indirect – between EL and turnover intentions has in fact emerged in several studies (Azanza et al., 2015; DeConinck, 2015; Demirtas and Akdogan, 2015; Lindblom et al., 2015; Babalola et al., 2016).

### Theoretical Background and Hypotheses Development

#### NPOs and Volunteers

Attracting and retaining volunteers is one of the main tasks as well as one of the biggest challenge of the management of NPOs (Salamon et al., 2013; Salamon, 2015). In fact, although the number of people deciding to engage in volunteering activities has grown in recent years, many organizations struggle to maintain volunteers’ commitment for a long time (Garner and Garner, 2011). Dropout rates of volunteers are consistently high across different countries, activities and types of organizations. Estimates about volunteers’ dropout show that at least one third per year leave the organization they are volunteering for (Chacón et al., 2007; Garner and Garner, 2011). Volunteers’ turnover is a serious threat for NPOs, who rely completely, or in great part, on volunteers for delivering their services, as well as for carrying out organizational activities, such as fundraising, administration, and other supporting duties (e.g., preparing newsletters) (Wymer and Starnes, 2001).

Given its positive links with volunteers’ intention to remain in the same organization, EL might thus help to counteract the negative trend of volunteer turnover as it is positively linked to the intention to remain in the same organization. Moreover, EL has also emerged to be positively linked with affective organizational commitment and job satisfaction. These are among the most successful factors of performance in organizations as well as in the management of volunteers (Reed et al., 2006; Bang et al., 2013; Neubert and Halbesleben, 2015).

Affective organizational commitment is defined as an individual’s positive emotional attachment to and involvement in the organization where s/he works (Mowday et al., 1979; Allen and Meyer, 1996; Solinger et al., 2008). In all kinds of organizations, no matter whether they are for profit or non-profit, organizational affective commitment has proven to be strongly related with the internalization of organizational values, dedication and loyalty, as well as with the alignment with the organization’s goals (Beck and Wilson, 2000; Patrick and Sonia, 2012).

The literature on affective commitment also highlights the strong link of this construct with an individual’s satisfaction with the work performed. Several studies in fact point out that high levels of satisfaction are fundamental to generate high levels of affective commitment (Lok and Crawford, 2001; Kim and Brymer, 2011; Bang et al., 2013; Vecina and Chacón, 2013b; Tahernejad et al., 2015; Chordiya et al., 2017).

Job satisfaction is described as “how people feel about their jobs and different aspects of their jobs. It is the extent to which people like or dislike their jobs” (Spector, 1997, p. 2). This definition posits that job satisfaction is a multi-dimensional construct, requiring to be measured and assessed from multiple viewpoints. Individuals have different expectations and ways of assessing their job, therefore a worker (or a volunteer) might be satisfied with some aspects of his/her work, while feeling neutral or dissatisfied with others. Moreover, each individual may assess differently the weight of each facet of a job (Locke, 1976; Schmidt, 2007; Falco et al., 2008). With regards to volunteer satisfaction, Vecina and colleagues (Vecina et al., 2009) identified three main dimensions: satisfaction with the motivation to volunteer, satisfaction with the duties and tasks performed, and satisfaction with the organization in which the volunteer operates. Satisfaction with motivation refers to the ability to gratify personal motivations, such as expressing values important to one’s self. Satisfaction with the tasks involves evaluating the tasks’ usefulness, their positive effects, and their ability to help the recipients. Whereas satisfaction with organizational management refers to the extent to which volunteers are satisfied by the way they are treated by the organization with which they are involved, which includes any training given and the recognition of the role played by the volunteers.

Overall, the main difference between the two concepts is that “affective commitment emphasizes the attachment to the organization, including its goals and values, satisfaction emphasizes the specific task environment where an individual performs his or her duties” (Mowday et al., 1979, p. 226). Affective commitment is thus an attitude that develops over time and therefore tends to be more stable than satisfaction (Yıldız and Şimşek, 2016). Satisfaction is in fact more linked to the assessment of the actual experience of the work performed, and is thus more prone to change over time (Porter et al., 1974).

#### Objectives and Purpose of the Study

**FIGURE 1:**
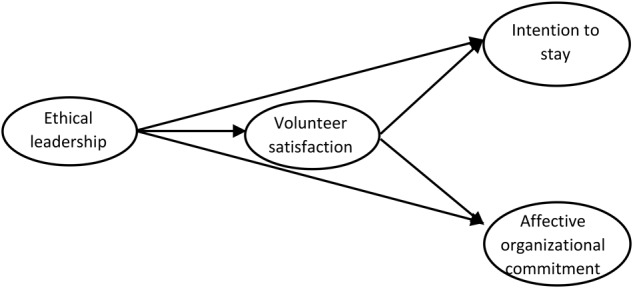
Hypothesized model.

This study aims to investigate the role played by EL in NPOs. More precisely, this study aims to first investigate the effect of EL on volunteers’ satisfaction, as well as on their affective organizational commitment and intention to stay in the same NPO. Then, consistent with the literature on the impact of ethical leaders on their followers, and in light of the functional theory of Clary and colleagues (Clary et al., 1998) the papers seeks to understand how volunteers’ satisfaction may play a moderating role in explaining how EL is related to volunteers’ affective commitment with the organization and their intention to stay in the same NPO. In light of the Theory of Planned Behavior (Ajzen and Madden, 1986; Ajzen, 1991), we assume that volunteers’ intention to stay is a strong predictor of the actual behavior of remaining to work as a volunteer in the same NPO. Figure 1 highlights the hypothesized model. To the authors’ knowledge no previous study has yet addressed this relationship and, more in general, the effects of EL on NPOs management. The study aims to address this gap as the authors believe that it is of both theoretical and empirical importance to develop knowledge on the effects of EL on NPOs, namely on volunteer’s satisfaction, intention to stay, and affective commitment. On one hand, this issue is relevant for scholars to further deepen their knowledge on EL and its effects on the followers, as argued by Van Knippenberg and Sitkin (2013). Moreover, it is also important to further develop the management theory for NPOs, in order to understand how organizational factors such as EL are playing a major role on the fruitful management of volunteers as well as on the prevention of their turnover (McMurray et al., 2010). On the other, from the practitioners and managers’ perspective this issue is also relevant, as understanding the relationship between EL and volunteers’ turnover might offer valuable insights on how to prevent volunteers’ dropout and to promote their organizational commitment and job satisfaction (do Nascimento et al., 2018). The latter two are in fact both strong antecedents of performance and organizational success (Miller et al., 1990; Gilbert et al., 2017).

This paper is divided into four main parts. The first part deals with the theoretical background and the development of the study’s hypotheses, and is divided into three main sub-sections. In the first sub-section, we analyze the construct as well the effects of EL on volunteers, in light of the Social Exchange Theory and the Social Learning Theory (Brown and Treviño, 2006). We hypothesize that EL has a direct effect on volunteers in terms of job satisfaction, intention to stay, and affective organizational commitment.

In the following sub-section, we examine volunteers’ behavior as an output of both individual motivations to volunteer and their actual experience of volunteering. Using the Theory of Planned Behavior (Ajzen, 1991) and the Functional Theory developed by Clary et al. (1998), we highlight that when volunteers find a positive balance between their expectations and the costs of their actual volunteering experience, they develop satisfaction for the work performed. This satisfaction, in turn, develops a stronger relationship with the NPO where they operate, leading thus to a greater intention to stay and affective commitment. We therefore draw the hypothesis that satisfaction with the actual experience of volunteering has a predictive power in generating volunteers’ intention to stay and their affective organizational commitment.

In the third sub-section, we argue that EL leverages volunteers’ satisfaction, which, in turn, is an antecedent of volunteers’ intention to stay and their affective organizational commitment. Thus, we hypothesize a mediating role of volunteers’ satisfaction in the relationship between EL and volunteers’ intention to stay, on one hand, and affective organizational commitment, on the other.

The second part of the study then presents the methodology adopted for the study. The third and fourth parts respectively present the results of the data analyzed, and discuss the findings by comparing the final results from the study with the model hypothesized.

#### Ethical Leadership and Its Impact on Volunteers

Ethical leadership is defined as “the demonstration of normatively appropriate conduct through personal actions and interpersonal relationships and the promotion of such conduct through two-way communication” (Brown et al., 2005, p. 120).

It has to be noted that, unlike the philosophical approach, which defines EL from a normative approach (that is describing “What an EL must do”), the above definition of EL adopts a descriptive approach, aimed at identifying behaviors, antecedents, and consequences of EL (Brown et al., 2005). Specifically, the first part of the definition refers to the fact that the leader, in order to be legitimized as a credible figure, should adopt behavioral patterns that are seen by his/her followers as appropriate from a normative and ethical point of view. The second part of the definition emphasizes how the ethical leader focuses his/her attention on the ethical aspects raised by the work of the organization and its members, speaking explicitly about it with the employees. It underlines that the ethical leader also establishes procedures and interpersonal relationships based on fairness, which allow the followers to express their point of view on ethical issues (Brown et al., 2005). This definition also implies that the leader establishes ethical standards, rewarding the behaviors of those who follow these rules, norms and principles, whilst intervening on those who do not follow these standards (Treviño and Ball, 1992; Gini, 1998; Treviño et al., 2003).

The last part of the definition proposed by Brown and Treviño refers to the decision-making process. In particular it highlights that ethical leaders should take fair decisions, aware of the consequences that these entail, and in light of the ethical standards that they themselves have adopted. These choices can therefore be observed from the outside and emulated by other members of the organization (Burns, 1978; Howell and Avolio, 1992; Bass, 1999).

With regards to the concept’s origins, it is worth highlighting that EL finds its theoretical foundation in the Social Exchange Theory (Blau, 1964) and in the Social Learning Theory (Bandura, 1986).

The Theory of Social Exchange posits that individuals who decide to engage in volunteering also assess knowingly and rationally the activities they perform, taking into account the benefits and costs that such engagement entails. Thus, if the costs of volunteering are positively counterbalanced by the received benefits, individuals are likely to maintain their commitment. Benefits can be both intrinsic and extrinsic (Cnaan and Amrofell, 1994) such as: social approval (Haski-Leventhal and Bargal, 2008), opportunities to receive training and develop their professionalism (Zakour, 1994), and raising one’s mental well-being (Wilson, 2000; Yanay and Yanay, 2008). As for the costs of voluntary commitment, these include the reduction of free time (Omoto and Snyder, 1993), psychological fatigue and stress (Capner and Caltabiano, 1993; Haski-Leventhal and Meijs, 2011), and negative social reactions, in the event that one chooses to operate with organizations whose beneficiaries constitute controversial social categories, such as those working with detainees (Omoto and Snyder, 1993).

The Theory of Social Exchange explains how ethical leaders shape the behavior of employees through social exchange processes. In fact, according to this theory, employees who receive care and attention from their superiors will be inclined to return such attention to their colleagues, clients, and managers. As a result of his/her behavior, the ethical leader is rewarded with positive organizational behaviors by making his/her followers experience justice, trust, and fairness (Brown et al., 2005).

The Social Learning Theory (Bandura, 1986) instead highlights that leaders exercise their influence over the ethical conduct of their followers through role modeling – i.e., through the followers’ direct observation, imitation, and identification with the leader’s behavior. To activate this modeling process, the leader must be an attractive and believable role model for his/her followers.

The ethical leader’s influence can also be exerted through vicarious learning: followers learn which behaviors are desirable and which are to avoid by observing the ways in which other members of the organization are rewarded or “warned” by the leader him/herself. Leaders who pay attention to integrity, and display interest and fairness toward their collaborators become an attractive role model, amplified by the status and power that leaders normally enjoy (Dinc and Aydemir, 2014). Importantly, subordinates who see in their ethical leader a role model are more likely to develop higher levels of job satisfaction, due to the trust and respect they have toward the leader (Ogunfowora, 2014).

Overall, Neubert and colleagues found out that individuals tend to be more satisfied with the work they perform and more committed to their organization when working in an environment characterized by ethical conduct, honesty, concern for others, and interpersonal fairness; in other words, a virtuous organization is perceived as ethical (Neubert et al., 2009; Kim and Brymer, 2011).

In fact, employees’ perception of their supervisor’s behavior plays a strong role in shaping their attitudes toward their workplace (Bonner et al., 2016; De Carlo et al., 2016). A leader’s ethical dimension has constantly proven to be an important predictor of high levels of affective organizational commitment (Valentine and Barnett, 2003; Brown and Treviño, 2006; Neubert et al., 2009; Yates, 2014; Wang and Xu, 2017). Finally, working in an ethical context may enable individuals to experience pride in their activity, leading to lower levels of intention to quit, therefore preventing high rates of turnover within an organization (Pettijohn et al., 2008).

It is possible to hypothesize that the above-mentioned effects of EL on paid workers might well be found among individuals who work on a voluntary basis for an NPO. In fact, previous studies have shown that leadership style among NPOs may predict followers’ outcomes such as volunteers’ satisfaction (Dwyer et al., 2013; Oostlander et al., 2014), as well as volunteers’ affective organizational commitment (Cha et al., 2011; Carder, 2012; Öztekin et al., 2015; Ajobiewe, 2017) and intention to stay (Bang, 2011; Schneider and George, 2011).

As a result of the above considerations of EL’s outcomes, the following hypotheses were developed:

Hypothesis 1a: EL is positively related to volunteers’ satisfaction.
Hypothesis 1b: EL is positively related to volunteers’ affective organizational commitment.
Hypothesis 1c: EL is positively related to the intention to stay in the same volunteer organization.

#### Volunteers’ Behavior and Volunteers’ Satisfaction

Volunteering is a behavior characterized by four factors that distinguishes it from other prosocial actions (Omoto and Snyder, 1995; Penner, 2004): it is a planned behavior; it is a long-term behavior; it is a non-obligatory help/support behavior; and it takes place within an organization.

Studies on volunteers’ behavior are grounded on the theory of planned action, which postulates that people make choices in a rational way, based on the information accessible to them (Fishbein and Ajzen, 1975). More precisely, the immediate antecedent of an action is the intention to implement it. In turn, the intention has three antecedents: the attitude (individual’s beliefs that the volunteering behavior leads to positive or negative outcomes); the subjective norms (the perceived social pressure – in both negative and positive terms – toward the adoption of the volunteering behavior and the individual’s desire to stay comply with such pressure); the perception of behavioral control (individual’s beliefs about the presence or absence of requisite resources and opportunities to be engaged in the volunteering behavior) (Ajzen, 1991). Since volunteering is a long-term planned behavior, it is assumed that the intention to volunteer predicts subsequent actual volunteer behavior (Warburton and Terry, 2000).

The theory of planned behavior has therefore been used to predict the likelihood that individuals decide to join a volunteer organization, (Warburton and Terry, 2000; Okun and Sloane, 2002), the amount of time spent volunteering (Greenslade and White, 2005) and their intention to remain volunteering in the same organization (Vecina and Chacón, 2013a).

The Functional Theory of Clary and colleagues (Clary et al., 1998), it explains the actual length of the volunteering behavior, through the satisfaction of the volunteers’ individual needs. In fact, according to this theory, individuals engage in voluntary activities because it allows them to satisfy different needs, such as: values (volunteering to express one own’s values); understanding (volunteering to understand better the world or to make use of personal knowledge, skills, and abilities otherwise unused); social (volunteering to strengthen his/her social relationships); career (volunteering to develop of skills and knowledge useful to one’s own professional path); protective (volunteering to protect the self from negative feelings, such as guilt, or to address personal problems); enhancement (volunteering to enhance self-esteem) (Clary et al., 1998).

It is therefore clear that “different individuals can participate in the same volunteer work for very different reasons; … volunteering can satisfy different motives for the same individuals at different times…” (Finkelstein et al., 2005, p. 404). Moreover, it is thus important to understand that people continue volunteering so far as their functional motives are satisfied by the organization they serve (Clary and Snyder, 1999).

In this regard, some authors point out that the motivations involved in the decision to become a volunteer are different from those that influence the decision to maintain this choice over time and to retain its presence within an organization (Winniford et al., 1995; Marta and Pozzi, 2008; Francis and Jones, 2012). In fact, since volunteering is a long-term planned behavior, within a dynamic process where several factors intervene, it is the experience of volunteering itself that modifies the initial motivations of volunteers (Omoto and Snyder, 1995; Penner, 2004).

Over time, the variables that come into play in determining a choice toward voluntary work change, or take on a different weight from those that determine the initial choice. According to McCurley and Lynch (1996), the first 6 months of volunteering are the most critical phase in shaping the decision to stay in an organization. In fact, the highest turn-over occurs in the second half of the year of volunteering.

Starting from this data, Wymer and Starnes (2001) have developed a model that includes two life cycles of volunteering. More in details: the volunteers start to provide their service in a phase defined as “honeymoon,” which is characterized by enthusiasm, by the desire to engage in their own voluntary work, and by gratification and satisfaction for the work done. After this stage another one takes over, where the knowledge of the organization in which it operates is less idealized and more realistic, because it is based on the direct experience carried out up to then within the organization itself. The phase of the “post-honeymoon” is therefore inevitably partly a phase of disillusionment, where the idealization of the first 6 months gives way to the knowledge and awareness of the critical aspects of the organization. If not counterbalanced by other positive factors, these realizations risk leading a volunteer to leave the organization (McCurley and Lynch, 1996). This means that in the relationship with the organization, the volunteers must develop a sense of satisfaction for the work done, as well as organizational commitment, to decide to stay in the same organization.

The possibility of having high levels of satisfaction with their volunteering activities is important in determining the amount of time individuals spend volunteering and the duration of that commitment, as well as in fueling their personal growth and self-esteem. Satisfaction, in fact, allows volunteers to feel compensated for the commitment given in situations of great emotional burden without necessarily receiving any extrinsic reward. This assumption has been confirmed by the studies of Chacón et al. (2007), who developed a three-phase model of volunteering, which refers to factors that predict the intention to continue to serve in the same organization in the short, medium, and long-term. In particular, in this model the satisfaction of the work done in the first 6 months in an organization constitutes the strongest predictor of the intention to remain in the same organization. In the second phase, the strongest predictive factor is organizational commitment. Finally, in the third phase the most significant predictive factor becomes role identity (Chacón et al., 2007). In this second model, volunteer satisfaction is linked to organizational commitment, and volunteers’ intention to stay, too. Intention to stay, according to the Theory of Planned Behavior, is in fact a strong predictor of the actual behavior (Grube and Piliavin, 2000; Vecina et al., 2009).

Both the above models of volunteering (McCurley and Lynch, 1996) assume that the management of volunteers plays a pivotal role in developing motivations and attitudes, which differ from the initial ones that led an individual to volunteer in the first place. This change can overcome at least partially or completely the distance between volunteers’ initial expectations and their actual organizational experiences, which, in turn, may generate satisfaction for the work done. In other words, these approaches underline the relevance of the dynamic interaction between the volunteers and the organization and its management.

Finally, according to different studies, volunteers’ satisfaction emerged to be a crucial antecedent of volunteers’ intention to stay as well as their organizational commitment, which develops and consolidates mainly after the first phase of the volunteering cycle (Bang et al., 2013; Neubert and Halbesleben, 2015).

As a result of the above considerations, the following hypotheses were developed:

Hypothesis 2a: Volunteers’ satisfaction is positively related to volunteers’ intention to stay.
Hypothesis 2b: Volunteers’ satisfaction is positively related to volunteers’ affective organizational commitment.

#### The Mediating Role of Volunteer Satisfaction

Volunteers’ behavior has been observed mainly from the perspective of personality and dispositional traits and much less from the perspective of their management by the organization they volunteer for Penner (2002) and Vecina and Chacón (2013b). Personality traits have received much attention since they are quite stable (Claxton-Oldfield et al., 2013; Falvo et al., 2013, Falco et al., 2017), and therefore have been regarded as predictive of volunteering behaviors (Penner and Finkelstein, 1998; White et al., 2017). More precisely, a number of studies used the five-factor model of personality and found that conscientiousness and agreeableness are relevant to volunteering (e.g., Carlo et al., 2005; Claxton-Oldfield and Banzen, 2010; Omoto et al., 2010). Previously, Penner (2002) found other-oriented empathy and helpfulness as the two main traits of volunteers. Other personality traits emerged as predicting volunteer behaviors are: resilience, extraversion, self-efficacy, low levels of neuroticism (Carlo et al., 2005; Matsuba et al., 2007; Okun et al., 2007). Value orientation also emerged as predictive of volunteerism, and chief amongst these were altruistic and religious values (Perry et al., 2008; Einolf, 2011). Finally, as far as personal and dispositional traits are concerned, the relevance of personality-organization fit also emerged as a key factor in volunteering. That is, the congruence between the personality traits of volunteers and the organizational environment may play a relevant role in determining the intention to leave the organization itself, since it is related to job satisfaction and organizational commitment, which are important antecedents of the intention to stay in the same organization (Miller et al., 1990; Van Vianen et al., 2008).

These studies clearly show how personality and dispositional traits, though relevant, cannot exhaustively explain volunteer turnover and organizational commitment. The limit of this personality-based approach lies in the fact that volunteering activities do not occur in a vacuum. They take place mostly within organizational contexts, that is among a dynamic relationship between the individuals and the organization itself (Penner, 2004).

Indeed it is important to underscore that organizational factors also play a crucial role in determining volunteers’ intention to quit, interacting with dispositional and personality characteristics (Egri and Herman, 2000; Willems et al., 2012). From the NPOs’ point of view, it is important to understand which factors related to the organization’s management are to be followed more accurately, since they have direct control over them, unlike the volunteers’ personality traits. Moreover, deepening the knowledge about the organizational factors that lead to lower intentions to quit and higher affective organizational commitment may further help to develop management theory on NPOs.

The existing literature highlights that important organizational factors in determining the intention of volunteers to quit the organization include: dissatisfaction with the work carried out (Vecina et al., 2009; Haivas et al., 2013); lack of training (Skoglund, 2006); role ambiguity (Allen and Mueller, 2013); lack of organizational support (McBride et al., 2012); the lack of recognition for the work done (Cohen-Callow, 2008); inadequate supervision (McBride et al., 2012) or, more generally, inadequate leadership (Willems et al., 2012).

From this overall picture, it emerges that the leadership of NPOs plays a crucial role in generating a positive and fruitful relationship between the NPO and the volunteers. More specifically, leaders have the power to shape the perception of the organization by the followers and, therefore, followers’ attitudes toward the work they perform. Thus, the observation of styles and modes of leadership can offer important suggestions for effective volunteer management, in terms of preventing and countering the turn-over of volunteers and promoting their commitment (Pierro et al., 1995).

The effect of EL on organizational outcomes has not yet been explored among NPOs. However, a number of studies carried out among organizations belonging to the for-profit and public sectors have proven not only the direct effects of EL on organization’s outcomes, but also the relevance of different mediating factors in the relationship between EL and followers’ outcomes, such as their intention to stay and affective commitment. More specifically, empirical studies have highlighted the mediating role of the ethical climate as well as the work-related stress between EL and employees’ intention to stay and their affective commitment (Mulki et al., 2008; Neubert et al., 2009; Kim and Brymer, 2011; Elçi et al., 2012; Yang, 2014; Demirtas and Akdogan, 2015; Tahernejad et al., 2015; Bedi et al., 2016; Tu et al., 2017; Wang and Xu, 2017).

A number of other studies also found evidence for the mediating role played by job satisfaction between the organization’s ethical dimensions on one side and the employees’ affective commitment and turnover on the other (Pettijohn et al., 2008; Kim and Brymer, 2011; Vecina and Chacón, 2013b; Palanski et al., 2014; Tahernejad et al., 2015). However, none of the studies carried out have until now dealt with NPOs.

It is then possible to hypothesize that the volunteers’ satisfaction plays a key role in generating positive outcomes of EL, by mediating the relationship between EL and the volunteers’ intention to stay, on one hand, and their affective commitment to the organization, on the other.

We assumed that, on the basis of a strong and positive role model proposed at both individual and organizational level, the specific pattern of EL behavior would enhance volunteers’ satisfaction, through the fulfillment of their expectations. Volunteers’ satisfaction in turn develops their loyalty to the organization and strengthens their relationship with the organization itself, fostering their willingness to stay and their affective commitment.

As a result the following hypotheses were postulated:

Hypothesis 3a: Volunteers’ satisfaction mediates the positive relationship between EL and volunteers’ intention to stay.
Hypothesis 3b: Volunteers’ satisfaction mediates the positive relationship between EL and volunteers’ affective organizational commitment.

## Materials and Methods

### Sample and Procedure

Five NPOs operating in Italy in the field of social work were contacted firstly by telephone and subsequently by email, in order to invite them to take part in the study. All of them were volunteer organization – i.e., the majority of their members were volunteers. Four accepted to allow their volunteers to be contacted by researchers, during one of their meetings. During these meetings researchers gave both written and oral explanations about the responsibility, the purpose and the procedure of the study, the content of the questionnaire that would be administered, as well as the anonymity and the confidentiality of the data collected. Researchers also provided further information or clarification, if required. All participants gave their written informed consent before the administration of the questionnaire, in accordance with the Declaration of Helsinki. The study was carried out in accordance the rules of AIP (Associazione Italiana di Psicologia – Italian Association of Psychology), according to which there was no need for previous ethics approval, since it would not deal with animals or vulnerable groups, or would involve risk for the well-being of participants, or use biomedical devices or invasive investigation tools. Our study did not need ethics approval, according to our national regulations as well as to the Ethics Committee of the LUMSA University. Participants filled individually the questionnaire, on a voluntary basis and received no money for their participation in the study. They completed the questionnaire in approximately 15 min. The volunteers reached were 212, of whom 198 accepted to fill the questionnaire. Respondents were all engaged in volunteering activities in organized contexts. The average age of the participants was of 38.05 years (minimum 14, maximum 76, DS = 16.37). 52.9% of the sample were women and 47.1% men. With regards to educational levels, 31.3% of participants completed only elementary or middle school, 39.4% held a high school diploma or a vocational qualification, 11.6% hold an undergraduate degree, 10.6% held a master’s degree or equivalent, and 7.1% held a doctorate or a further kind of specialization. Students represented the highest proportion of participants (26.4%), followed by employees (21.8%). In addition, the percentage of free professionals volunteering was the same as that of pensioners (12.7%), exceeding that of housewives (9.1%), and those who were unemployed (8.1%). Some participants were also workmen (5.1%), executives or middle managers (2%), teachers (1.5%), and shopkeepers (0.5%). Most of the participants had been working for the same volunteer organization for more than 12 months (69.7%), followed by 18.7% who had been working for a period ranging between 6 and 12 months, and a minority (11.6%) that had been working for less than 6 months. For 66.1% of participants, their current volunteering experience was the first organized volunteer context in which they have worked, whereas 33.9% of participants have already had previous experience in other organizations. Among the latter group, the average amount of time they had worked in their organization was of 42.56 months (minimum 1, maximum 249, DS = 63.08). Overall, the average weekly dedication to volunteering amongst the participants was of 12.34 h (minimum 2, maximum 70, DS = 12.12).

### Measures

#### Ethical Leadership

The Ethical Leadership Scale was used (Brown et al., 2005). The instrument, adapted to the context of volunteering, consists of 10 items assessed using a 5-point Likert scale (1 = very disagree, 5 = very much agree). An example of an item is the following: “My supervisor sets an example of how to do things the right way in terms of ethics.” Cronbach’s alpha of this scale is of 0.93.

#### Volunteer Satisfaction

The Volunteer Satisfaction Index was used to measure the satisfaction of volunteers (Vecina et al., 2009). The scale includes three sub-scales, assessed using a 10-point Likert scale (1 = I totally disagree; 10 = I absolutely agree). The first consists of 6 items, focusing on the extent to which volunteering activities meet some of the major motivations previously identified by Clary and colleagues; namely: value, understanding, social, career, protective, enhancement (Clary et al., 1998). An example of an item in this sub-scale is “My volunteering allows me to express my personal values.” Cronbach’s alpha of this dimension is of 0.89. The second sub-scale is composed of 5 items, which analyze satisfaction with the tasks performed. An example of an item in this sub-scale is: “I can easily find out if I’m doing my tasks well as I do my volunteer work.” Cronbach’s alpha of this dimension is of 0.92. The last sub-scale is made up of 7 items, relating to the satisfaction with organizational management. An example of an item in this sub-scale is the following: “I am satisfied with the mechanisms in place to deal with problems encountered by volunteers during their work.” Cronbach’s alpha of this dimension is of 0.73. Cronbach’s alpha for the whole scale is equivalent to 0.90.

#### Affective Organizational Commitment

The tool proposed by Allen and Meyer (1990) was used to measure affective organizational commitment. The scale was adapted to the volunteer context and consists of 6 items assessed using a 5-point Likert scale (1 = very disagree; 5 = very much agree). An example of an item in this scale is the following: “I really feel like that’s the organization’s problems.” Cronbach’s alpha of the scale is equivalent to 0.92.

#### Intention to Stay

A single item was used (“If there are no reasons for force majeure, I think I will volunteer in this organization”) to measure a volunteer’s intention to stay, which included 3 categories of response (“For the next 6 months,” “6–12 months,” “Over 12 Months”).

The questionnaire collected socio-demographic information about participants, namely: age, sex, type of work, level of education, length of their volunteer works, intention to volunteer at least in the following 6, 12 and more than 12 months.

## Results

Means, standard deviations, and correlations are presented in Table 1.

**Table 1:** Means, standard deviations, and correlations.

Variable	*M*	*SD*	1	2	3	4
Ethical leadership	4.10	0.74	1.00			
Volunteer satisfaction index	7.79	1.59	0.42^∗∗^	1.00		
Intention to stay	–	–	0.17^∗^	0.20^∗∗^	1.00	
Affective organizational commitment	4.08	0.81	0.52^∗∗^	0.48^∗∗^	0.23^∗∗^	1.00

*n = 198, ^∗^*p* < 0.05; ^∗∗^*p* < 0.01.*

The data was processed using structural equation models (SEM) in two steps: verification of the measurement model and the structural test. The analyses were carried out using the Lisrel 8.80 software (Jöreskog and Sörbom, 2006) with the Robust Maximum Likelihood method. We estimate the fit of our measurement model in terms of χ2, Non-normed Fit Index (NNFI), Comparative Fit Index (CFI), Root Mean Square Error of Approximation (RMSEA) and Standardized Root Mean Square Residual (SRMR). The model has a good adaptation to the data, since χ2 = 5.69, *p* = 0.46, NNFI = 1.01, CFI = 1.00, RMSEA = 0.00 and SRMR = 0.10 (Schermelleh-Engel et al., 2003).

The hypothesized structural model was then tested (Figure 1). Based on the literature, EL is the independent variable (Brown et al., 2005; Brown and Treviño, 2006; Kim and Brymer, 2011; Qin et al., 2014; Lin and Liu, 2017), the volunteer is the mediator (Kim and Brymer, 2011; Vecina and Chacón, 2013b; Tahernejad et al., 2015), while the intention to stay in the same organization and the affective commitment are dependent variables (Jaramillo et al., 2006; Pettijohn et al., 2008; Vecina et al., 2012; Vecina and Chacón, 2013b; Palanski et al., 2014; Demirtas and Akdogan, 2015; Tahernejad et al., 2015).

To verify the significance of the two specific indirect effects of EL on the intention to remain and affective organizational commitment, 95 percent asymmetric confidence intervals were considered, based on the distribution of the multiplication term (PRODCLIN; MacKinnon et al., 2007; MacKinnon, 2008). If the confidence interval does not contain zero, the significance of mediation is supported (MacKinnon et al., 2012).

First, the hypothesized structural model was estimated (Figure 1). Fit indices show a good adaptation to data, considering χ2 = 5.69, *p* = 0.46; NNFI = 1.01 CFI = 1.00; RMSEA = 0.00. In the model, EL is positively associated with satisfaction for the work done (γ = 0.34, *p* < 0.01), therefore hypothesis 1a is supported. In addition, satisfaction with the work done is positively associated with the intention to remain in the same organization (β = 0.27, *p* < 0.05) and the affective commitment (β = 0.38, *p* < 0.01), confirming hypotheses 2a and 2b, respectively.

The asymmetric confidence interval for the relationship between EL and intention to remain, through satisfaction, does not contain zero; the unconventional estimate is.09, 95% CI [0.003,0.181]. Therefore, we can conclude that satisfaction for the work mediates the relationship between EL and intention to remain, confirming hypothesis 3a. In addition, when controlling the effect of satisfaction, the positive relationship between EL and intention to remain is not significant (γ = 0.09, *p* > 0.05), we conclude that such mediation is total; therefore, hypothesis 1c is not confirmed.

Furthermore, also the asymmetric confidence interval for the relationship between EL and affective organizational commitment through satisfaction does not contain zero; the unconventional estimate is 0.13, 95% CI [0.03,0.25]. We can thus conclude that satisfaction for work mediates the relationship between EL and affective organizational commitment, confirming hypothesis 3b. Moreover, since EL is positively associated with the effective organizational commitment by controlling the effect of satisfaction (γ = 0.26, *p* < 0.001), we conclude that such mediation is partial.

**FIGURE 2:**
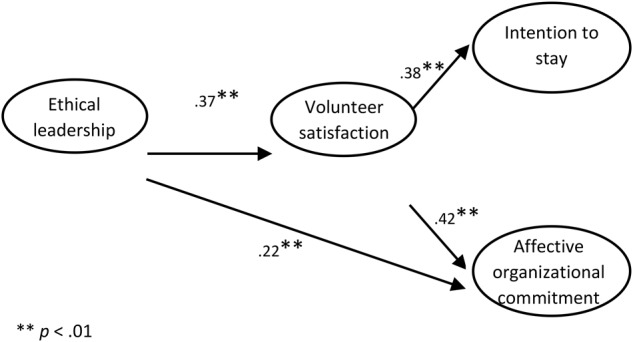
Final model.

Subsequently, in order to obtain a more parsimonious solution, we estimated another model (Figure 2), where the meaningless path (which links EL and intention to stay) was set to zero. Since this bound model, considering fewer parameters, is more parsimonious and since it does not perform worse than the unconstrained model (Δχ2 = 1.37, *p* = 0.24), we believe it is preferable. Fit indices show good data adaptation, considering χ2 = 7.06, *p* = 0.42; NNFI = 1.00; CFI = 1.00; RMSEA = 0.01. In this model, EL is positively associated with satisfaction for the work done (γ = 0.37, *p* < 0.01), which in turn is positively associated both with the intention of remaining in the same organization (β = 0.38, *p* < 0.01), and to the effective organizational commitment (β = 0.42, *p* < 0.01). In addition, EL is directly and positively associated with the affective organizational commitment (γ = 0.22, *p* < 0.01), confirming hypothesis 1b.

## Discussion

### Discussion of Hypotheses and Results

The general aim of this study was to observe the effect of EL on volunteers, specifically by looking at its effect on their job satisfaction, their intention to stay in the same organization, and their affective organizational commitment.

With regards to volunteer satisfaction’s mediation role, the results confirm this role in the relationships between the variables studied. In particular, EL was found to be positively associated with both volunteers’ intention of stay and their affective organizational commitment. The relationship between EL and affective commitment is explained both directly and indirectly, through the mediation of job satisfaction. Unlike what was hypothesized, the relationship between EL and volunteers’ intention of stay is fully mediated by job satisfaction, and EL has no direct effect on the intention to stay.

This latter finding may be read in the light of a dynamic perspective, which assumes that, as time passes, the actual experiences of volunteers modifies the relationships among the variables considered. This means that the weight of the variables assessed may differ whether the focus of the assessment is on the intention to stay in the short or in the long run. In fact, satisfaction is a strong predictor of the intention to stay in the short run, but less relevant in the long run, where affective commitment is more relevant (Chacón et al., 2007). It would be interesting further develop this study, analyzing the data in the light of the volunteers’ intention to stay in the long and in the short run, in order to deepen the understanding of EL according these two variables. Moreover, according to the three-stages model of volunteering, the length of the service might generate differences in the relationship between the volunteers’ intention to stay and their affective organizational commitment.

Findings show also that EL has both a direct impact on volunteers’ affective organizational commitment, as it had already been found for employees of for-profit and public organization, as well as an indirect effect, through the mediation of volunteers’ satisfaction. The latter result can be read in the light of the three-stages model of volunteering, according to which volunteers’ satisfaction develops affective organizational commitment.

Findings of this study shed a light on the controversial role played by volunteers’ satisfaction in preventing their turnover. Indeed, the role of volunteers’ satisfaction has emerged as controversial, not only because the construct adopted to assess the volunteers’ satisfaction varies widely among different studies, but also because the length of the volunteers’ service modifies the satisfaction levels and its impact, reducing its predictive power (Chacón et al., 2007).

Findings of this study confirm the results of the research carried out by Chacón and colleagues, showing that volunteers’ satisfaction do play a role in preventing volunteers’ turnover. In fact, as Chacón and colleagues argued, “Satisfaction is not directly related to duration of service, but it is related to behavioral intention to remain, and this is the variable that most influences actual service duration” (Chacón et al., 2007, p. 639). However, even if the relevance of the volunteers’ satisfaction tends to diminish after the first phase of volunteering, the relevance of this factor still remains high.

### Findings’ Relevance for EL Theory

Firstly, the present study extends the current state of research on the influence exerted by EL in an organizational context never addressed previously; that is, the non-profit world. This study tested both its direct and indirect effect on volunteers’ outcomes, namely volunteers’ satisfaction, intention to stay and affective organizational commitment, showing the relevance of this relationship. Moreover, our findings further develop the knowledge on indirect effects of EL on followers, namely through a mediating mechanism, which is still a field of research not fully explored (Mayer et al., 2012; Eisenbeiss et al., 2015; Qian et al., 2017). Previous studies have shown the mediating role played between EL and employees’ outputs by a number of factors such as ethical climate, meaningfulness of work, self-efficacy and psychological capital, trust and leader-members exchange (Haller et al., 2018). Yet, till now the influence of EL on followers has not been observed from the perspective of volunteers’ satisfaction.

Secondly, findings of this study seem to confirm a previous study, where it was argued that EL is able to generate satisfaction for the work performed through a strong relational attachment, in the light of the attachment theory (Haller et al., 2018). Indeed, NPOs’ activities are continuously challenged to respond to old and new needs emerging from society and volunteers perform their work in an ever-changing social environment. The ethical leader can thus respond to the need for a safe base by his/her organization’s members. According to the attachment theory applied to adult relations (Bowlby, 1969, 1982), this security helps reduce work-related anxiety, which is in turn negatively associated with satisfaction for the work performed (Qin et al., 2014). Since the degree of satisfaction depends on the assessment of the context in which individuals are involved, it is possible to deduct that working in an ethical context enables them to experience pride in their activity, leading to higher levels of satisfaction (Pettijohn et al., 2008).

Thirdly, the present study adds a new insight into the power and the outputs of leadership among NPOs (Vélot, 2016). As noted before, EL has never been observed till now among these organization, though there are some evidences of the positive impact of ethical climate on these organizations, namely in terms of the interiorization of the workplace norms, values and behaviors by their members (Fenton and Inglis, 2007). However, previous studies on ethics among NPOs either adopted a normative approach or were limited to observe the difference of ethical climate and perceptions among the non-profit and for-profit world or among the different constituency of the same NPO (Rasmussen et al., 2003; Rothschild and Milofsky, 2006; McDonald et al., 2015). To the authors’ knowledge, none of the previous study dealt with the influencing process of EL on diverse follower outcomes, investigating from a NPOs management theory and practice perspective (Rowold et al., 2014).

### Findings’ Relevance for NPO Management

This study’s findings provide three important indications for how NPOs could address issues in their management of volunteers.

First of all, EL clearly emerged as a crucial factor in the successful management of volunteers, fueling their loyalty and commitment to the NPO where they work. This is a relevant point since over the last decades the NPOs have increasingly adopted business-like practices and approaches, in order to become more efficient and to successfully overcome the problems arising from the reduction of the governmental funds (Eikenberry and Kluver, 2004; Benevene and Cortini, 2010; Maier et al., 2016).

Organizational ethical behaviors of NPOs are heavily challenged by the need of balancing the NPOs’ social mission with their financial constraints. This requires NPOs to pay a strong attention on how to combine a market-based approach while still giving priority to their social raison d’être. Therefore, refocusing the organizational management in terms of EL may help to balance positively these two aspects (Dolnicar et al., 2008; Benevene et al., 2017).

In this regard, it has to be noted that introducing the focus on EL in any organization would require a proper attention from the management in terms of the content and the way in which the management communicates and endorses their choices. The positive role played by EL thus calls for more training to raise the awareness of the senior managers on this issue as well as on the actions and behaviors to be implemented. As Treviño and colleagues already noted, “To be perceived as an ethical leader, it is not enough to just be an ethical person. An executive ethical leader must also find ways to focus the organization’s attention on ethics and values and to infuse the organization with principles that will guide the actions of all employees” (Treviño et al., 2000, p.130). More in general, it is also to be noted that, attracting, selecting and above all retaining volunteers requires specific skills, on which managers have often received no specific training, except through their direct experience (Benevene and Cortini, 2010).

Second, from our findings volunteer’s satisfaction emerged as a crucial dimension, since it fully explains the effect of EL. Hence, in order to increase the rate of volunteers’ retention as well as their affective commitment, NPO managers should address effectively volunteers’ motivations in order to fulfill them. In fact, several studies have shown that the quality of the practices and tools adopted in the management of volunteers are a critical factor for the satisfaction and loyalty of volunteers (Skoglund, 2006; Cohen-Callow, 2008; McBride et al., 2012; Allen and Mueller, 2013).

Third, it should be highlighted that, under a strictly management perspective, these studies, including the present one, show that it is more is more productive to focus on organizational and managerial aspects than on volunteers’ personality traits, since the former are the most directly controlled by the organization.

Moreover, with regards to personality traits, a number of studies have highlighted that volunteering varies across one’s life course, reaching its peak in middle age, whilst at the same time showing some overall stable patterns (Marzana et al., 2015). In fact, as postulated by the continuity theory of aging, according to which people largely follow the habits they acquired at younger ages, “the majority of those who were volunteering at the beginning did not stop, and most of those who did not volunteer initially did not start later” (Lancee and Radl, 2014, p. 834).

Furthermore, with regards to those who start and then stop volunteering, it is well-known in the literature that some volunteers leave the organization in which they work due to important changes in their personal life, which intervene in preventing the continuation of their volunteering activities (such as: the reduction of available time available to be dedicated to volunteering, for work or family reasons, or the onset of health problems). These reasons are therefore not immediately to be considered as free and deliberate choices taken by individuals (Field and Johnson, 1993; Hustinx, 2010). Yet, those who leave for problems beyond their control constitute a small portion of the volunteers’ dropout rate and, generally speaking, those who quit their volunteer work are likely to go back to volunteerism at a later stage in their lives (Willems et al., 2012). This is a fact that pushes toward a deeper understanding of volunteers’ satisfaction and their intention to quit, particularly from the organizational, rather than the personality, point of view.

However, it must highlighted that the analysis of personality traits could in fact be a factor to be assessed during the process of selection of new volunteers, in order to identify these who are most likely to better fit with the organizational goals and activities. Yet, very few organizations have the resources to do so or can afford to reject any volunteer on the basis of his/her poor correspondence between organizational characteristics and personality traits (Van Vianen et al., 2008).

### Limitations and Future Directions of Research

This study has some limitations. First of all, there is a risk of bias due to the common method variance, which could be caused by the cross-sectional nature of the study and the data collection process, since all the variables studied were collected in the same way. In the future, designs that also include ethical evaluations as well as procedures that psychologically separate the relationships of dependent and independent variables will help reduce the risk of common method bias.

A second limitation of this study lies in the impossibility of definitely determining causal relationships. Indeed, while the study has explored the role of EL and volunteer satisfaction in promoting positive results, the cross-sectional data collected does not allow us to move forward, with regards to the causal relationships between the dimensions investigated. In the future, the implementation of longitudinal studies can shed light on the direction and the causal relationships between the variables studied. Another limitation is the limited generalizability of the results. The data of the present study were collected only in one country, namely in Italy. Hence it cannot be assumed that these findings could be considered as appropriate also for other context. Therefore it would be interesting to replicate this study in other countries, on a comparative basis. Finally, the scale we used to investigate the EL (Brown et al., 2005) has been widely validated, but has a one-dimensional structure. Future studies could use the multidimensional instrument, such the Ethical Leadership at Work questionnaire (ELW) (Kalshoven et al., 2011), in order to understand if all or some dimensions of EL have a deeper impact among NPOs volunteers. Furthermore, for the same reason, it would be useful replicate this study among employees of NPOs rather than just on volunteers.

## Discussion

### Discussion of Hypotheses and Results

The general aim of this study was to observe the effect of EL on volunteers, specifically by looking at its effect on their job satisfaction, their intention to stay in the same organization, and their affective organizational commitment.

With regards to volunteer satisfaction’s mediation role, the results confirm this role in the relationships between the variables studied. In particular, EL was found to be positively associated with both volunteers’ intention of stay and their affective organizational commitment. The relationship between EL and affective commitment is explained both directly and indirectly, through the mediation of job satisfaction. Unlike what was hypothesized, the relationship between EL and volunteers’ intention of stay is fully mediated by job satisfaction, and EL has no direct effect on the intention to stay.

This latter finding may be read in the light of a dynamic perspective, which assumes that, as time passes, the actual experiences of volunteers modifies the relationships among the variables considered. This means that the weight of the variables assessed may differ whether the focus of the assessment is on the intention to stay in the short or in the long run. In fact, satisfaction is a strong predictor of the intention to stay in the short run, but less relevant in the long run, where affective commitment is more relevant (Chacón et al., 2007). It would be interesting further develop this study, analyzing the data in the light of the volunteers’ intention to stay in the long and in the short run, in order to deepen the understanding of EL according these two variables. Moreover, according to the three-stages model of volunteering, the length of the service might generate differences in the relationship between the volunteers’ intention to stay and their affective organizational commitment.

Findings show also that EL has both a direct impact on volunteers’ affective organizational commitment, as it had already been found for employees of for-profit and public organization, as well as an indirect effect, through the mediation of volunteers’ satisfaction. The latter result can be read in the light of the three-stages model of volunteering, according to which volunteers’ satisfaction develops affective organizational commitment.

Findings of this study shed a light on the controversial role played by volunteers’ satisfaction in preventing their turnover. Indeed, the role of volunteers’ satisfaction has emerged as controversial, not only because the construct adopted to assess the volunteers’ satisfaction vary widely among different studies, but also because the length of the volunteers’ service modifies the satisfaction levels and its impact, reducing its predictive power (Chacón et al., 2007).

Findings of this study confirm the results of the research carried out by Chacón and colleagues, showing that volunteers’ satisfaction do play a role in preventing volunteers’ turnover. In fact, as Chacón and colleagues argued, “Satisfaction is not directly related to duration of service, but it is related to behavioral intention to remain, and this is the variable that most influences actual service duration” (Chacón et al., 2007, p. 639). However, even if the relevance of the volunteers’ satisfaction tends to diminish after the first phase of volunteering, the relevance of this factor still remains high.

### Findings’ Relevance for EL Theory

Firstly, the present study extends the current state of research on the influence exerted by EL in an organizational context never addressed previously; that is, the non-profit world. This study tested both its direct and indirect effect on volunteers’ outcomes, namely volunteers’ satisfaction, intention to stay and affective organizational commitment, showing the relevance of this relationship. Moreover, our findings further develop the knowledge on indirect effects of EL on followers, namely through a mediating mechanism, which is still a field of research not fully explored (Mayer et al., 2009; Eisenbeiss and van Knippenberg, 2015; Qian et al., 2017). Previous studies have shown the mediating role played between EL and employees’ outputs by a number of factors as ethical climate, meaningfulness of work, self-efficacy and psychological capital, trust and leader-members exchange (Haller et al., 2018). Yet, till now the influence of EL on followers has not been observed from the perspective of volunteers’ satisfaction.

Secondly, findings of this study seem to confirm a previous study, where it was argued that EL is able to generate satisfaction for the work performed through a strong relational attachment, in the light of the attachment theory (Haller et al., 2018). Indeed, NPOs’ activities are continuously challenged to respond to old and new needs emerging from society and volunteers perform their work in an ever-changing social environment. The ethical leader can thus respond to the need for a safe base by his/her organization’s members. According to the attachment theory applied to adult relations (Bowlby, 1969, 1982), this security helps reduce work-related anxiety, which is in turn negatively associated with satisfaction for the work performed (Qin et al., 2014). Since the degree of satisfaction depends on the assessment of the context in which individuals are involved, it is possible to deduct that working in an ethical context enables them to experience pride in their activity, leading to higher levels of satisfaction (Pettijohn et al., 2008).

Thirdly, the present study adds a new insight into the power and the outputs of leadership among NPOs (Vélot, 2016). As noted before, EL has never been observed till now among these organization, though there are some evidences of the positive impact of ethical climate on these organizations, namely in terms of the interiorization of the workplace norms, values and behaviors by their members (Fenton and Inglis, 2007). However, previous studies on ethics among NPOs either adopted a normative approach or were limited to observe the difference of ethical climate and perceptions among the non-profit and for-profit world or among the different constituency of the same NPO (Rasmussen et al., 2003; Rothschild and Milofsky, 2006; McDonald et al., 2015). To the authors’ knowledge, none of the previous study dealt with the influencing process of EL on diverse follower outcomes, investigating from a NPOs management theory and practice perspective (Rowold et al., 2014).

### Findings’ Relevance for NPO Management

This study’s findings provide three important indications for how NPOs could address issues in their management of volunteers.

First of all, EL clearly emerged as a crucial factor in the successful management of volunteers, fueling their loyalty and commitment to the NPO where they work. This is a relevant point since over the last decades the NPOs have increasingly adopted business-like practices and approaches, in order to become more efficient and to successfully overcome the problems arising from the reduction of the governmental funds (Eikenberry and Kluver, 2004; Benevene and Cortini, 2010; Maier et al., 2016; Benevene et al., 2018).

Organizational ethical behaviors of NPOs are heavily challenged by the need of balancing the NPOs’ social mission with their financial constraints. This requires NPOs to pay a strong attention on how to combine a market-based approach while still giving priority to their social raison d’être. Therefore, refocusing the organizational management in terms of EL may help to balance positively these two aspects (Dolnicar et al., 2008; Benevene et al., 2017).

In this regard, it has to be noted that introducing the focus on EL in any organization would require a proper attention from the management in terms of the content and the way in which the management communicates and endorses their choices. The positive role played by EL thus calls for more training to raise the awareness of the senior managers on this issue as well as on the actions and behaviors to be implemented. As Treviño and colleagues already noted, “To be perceived as an ethical leader, it is not enough to just be an ethical person. An executive ethical leader must also find ways to focus the organization’s attention on ethics and values and to infuse the organization with principles that will guide the actions of all employees” (Treviño et al., 2000, p.130). More in general, it is also to be noted that, attracting, selecting and above all retaining volunteers requires specific skills, on which managers have often received no specific training, except through their direct experience (Benevene and Cortini, 2010).

Second, from our findings volunteer’s satisfaction emerged as a crucial dimension, since it fully explains the effect of EL. Hence, in order to increase the rate of volunteers’ retention as well as their affective commitment, NPO managers should address effectively volunteers’ motivations in order to fulfill them. In fact, several studies have shown that the quality of the practices and tools adopted in the management of volunteers are a critical factor for the satisfaction and loyalty of volunteers (Skoglund, 2006; Cohen-Callow, 2008; McBride et al., 2012; Allen and Mueller, 2013).

Third, it should be highlighted that, under a strictly management perspective, these studies, including the present one, show that it is more is more productive to focus on organizational and managerial aspects than on volunteers’ personality traits, since the former are the most directly controlled by the organization.

Moreover, with regards to personality traits, a number of studies have highlighted that volunteering varies across one’s life course, reaching its peak in middle age, whilst at the same time showing some overall stable patterns. In fact, as postulated by the continuity theory of aging, according to which people largely follow the habits they acquired at younger ages, “the majority of those who were volunteering at the beginning did not stop, and most of those who did not volunteer initially did not start later” (Lancee, and Radl, 2014, p. 834).

Furthermore, with regards to those who start and then stop volunteering, it is well-known in the literature that some volunteers leave the organization in which they work due to important changes in their personal life, which intervene in preventing the continuation of their volunteering activities (such as: the reduction of available time available to be dedicated to volunteering, for work or family reasons, or the onset of health problems). These reasons are therefore not immediately to be considered as free and deliberate choices taken by individuals (Field and Johnson, 1993; Hustinx, 2010). Yet, those who leave for problems beyond their control constitute a small portion of the volunteers’ dropout rate and, generally speaking, those who quit their volunteer work are likely to go back to volunteerism at a later stage in their lives (Willems et al., 2012). This is a fact that pushes toward a deeper understanding of volunteers’ satisfaction and their intention to quit, particularly from the organizational, rather than the personality, point of view.

However, it must highlighted that the analysis of personality traits could in fact be a factor to be assessed during the process of selection of new volunteers, in order to identify these who are most likely to better fit with the organizational goals and activities. Yet, very few organizations have the resources to do so or can afford to reject any volunteer on the basis of his/her poor correspondence between organizational characteristics and personality traits (Van Vianen et al., 2008).

### Limitations and Future Directions of Research

This study has some limitations. First of all, there is a risk of bias due to the common method variance, which could be caused by the cross-sectional nature of the study and the data collection process, since all the variables studied were collected in the same way. In the future, designs that also include ethical evaluations as well as procedures that psychologically separate the relationships of dependent and independent variables will help reduce the risk of common method bias.

A second limitation of this study lies in the impossibility of definitely determining causal relationships. Indeed, while the study has explored the role of EL and volunteer satisfaction in promoting positive results, the cross-sectional data collected does not allow us to move forward, with regards to the causal relationships between the dimensions investigated. In the future, the implementation of longitudinal studies can shed light on the direction and the causal relationships between the variables studied. Another limitation is the limited generalizability of the results. The data of the present study were collected only in one country, namely in Italy. Hence it cannot be assumed that these findings could be considered as appropriate also for other context. Therefore it would be interesting to replicate this study in other countries, on a comparative basis. Finally, the scale we used to investigate the EL (Brown et al., 2005) has been widely validated, but has a one-dimensional structure. Future studies could use the multidimensional instrument, such the Ethical Leadership at Work questionnaire (ELW) (Kalshoven et al., 2011), in order to understand if all or some dimensions of EL have a deeper impact among NPOs volunteers. Furthermore, for the same reason, it would be useful replicate this study among employees of NPOs rather than just on volunteers.

## Author Contributions

PB developed the research project, with the contribution of MV, LDC, and ADC. AF carried out the data analysis, with the contribution of FC. FC contacted the volunteers, administered the questionnaire, and prepared the data set.

## Conflict of Interest Statement

The authors declare that the research was conducted in the absence of any commercial or financial relationships that could be construed as a potential conflict of interest.
